# Common Uses and Adverse Effects of Hyperbaric Oxygen Therapy in a Cohort of Small Animal Patients: A Retrospective Analysis of 2,792 Treatment Sessions

**DOI:** 10.3389/fvets.2021.764002

**Published:** 2021-11-24

**Authors:** Christina Montalbano, Caroline Kiorpes, Lindsay Elam, Erin Miscioscia, Justin Shmalberg

**Affiliations:** ^1^Department of Comparative, Diagnostic, and Population Medicine, College of Veterinary Medicine, University of Florida, Gainesville, FL, United States; ^2^Whole Animal Gym, Philadelphia, PA, United States; ^3^Department of Clinical Sciences, College of Veterinary Medicine and Biomedical Sciences, Colorado State University, Fort Collins, CO, United States

**Keywords:** hyperbaric oxygen therapy, integrative veterinary medicine, adverse events, oxygen toxicity, seizures

## Abstract

Hyperbaric oxygen therapy (HBOT) is commonly utilized for various human conditions with a low incidence of major adverse effects (0.002–0.035%). Despite growing use in veterinary patients, there remains a paucity of literature describing its use and associated complications. The purpose of this study was to report clinical use of HBOT in small animals and identify the rate of major adverse events at a university teaching hospital. Electronic medical records were searched for small animals receiving HBOT between November 2012 and February 2020. Data extracted from the medical records included signalment, treatment indication, and adverse events. Treatment sessions totaled 2,792 in 542 dogs, 24 cats, and 10 pocket pets and exotics. Common indications included neurologic injuries (50.4%), tissue healing (31.4%), control of oomycete infection (5.5%), neoplasia or post-radiation injury (5.4%), and various miscellaneous conditions (7.4%). Observed minor adverse events included agitation in two dogs and vomiting in three dogs. The most common major adverse event was central nervous system (CNS) oxygen toxicity in 19 dogs. Central nervous system oxygen toxicity, manifesting as focal or generalized seizures, occurred in 0.7% of treatment sessions, with increasing age (*p* = 0.01) and female sex (*p* = 0.01) identified as risk factors. One dog developed pulmonary edema following HBOT which is a reported adverse event in humans or may have been a manifestation of progression of the dog's underlying disease. No adverse events were noted in cats or other species. In conclusion, HBOT appeared safe across various indications, although oxygen toxicity affecting the CNS was higher than reports in humans. Future prospective, randomized, controlled trials should evaluate specific clinical indications and outcomes.

## Introduction

Hyperbaric oxygen therapy (HBOT) is a simple but effective treatment for a variety of medical conditions (1–3). Treatment involves the administration of 100% oxygen at increased pressure, typically performed between 1.5 and 3 times higher than standard air pressure at sea level. The subsequently elevated partial pressure of oxygen has a number of biochemical effects, such as increasing endogenous antioxidant production, stimulating peripheral vasoconstriction, modulating inflammation, increasing antimicrobial activity, and promoting angiogenesis (4). Hyperbaric oxygen therapy was initially used in humans to treat decompression sickness, with the first treatment performed by Behnke and Shaw in 1937 (5). Decompression sickness is still one of the most common and best researched indications for HBOT in humans, although the Undersea and Hyperbaric Medical Society lists a number of other accepted indications, including carbon monoxide poisoning, acute thermal burns, clostridial myositis, radionecrosis, acute ischemia, gas embolism, crush injury, intracranial abscesses, severe anemia, refractory osteomyelitis, compromised skin flaps and grafts, and necrotizing soft tissue infections (6).

Although HBOT is an accepted medical therapy in human medicine with research substantiating its efficacy, use in veterinary medicine is comparatively novel with conflicting results (7–9). Furthermore, there is a paucity of information regarding adverse events of HBOT in small animal veterinary patients. In human literature, HBOT appears to be a safe therapeutic modality, with most adverse events manifesting as transient discomfort (e.g., barotrauma affecting the middle or inner ear, nasal sinuses, or teeth and confinement anxiety) reported in 17% of patients in one study (10). Some effects may be more severe; central nervous system (CNS) oxygen toxicity resulting in a generalized seizure is reported in 0.002–0.035% of human patients (10–13). Oxygen toxicity can also contribute to lung pathology (10, 14, 15). Recognizing signs of discomfort associated with barotrauma in veterinary patients is challenging, but may manifest as head shaking or yawning. Focal or generalized seizures are more readily recognized in veterinary patients. Potential adverse effects in veterinary patients are poorly documented due to uncommon occurrences and have unknown consequences (16, 17). In people, oxygen toxicity induced seizures do not appear to cause adverse sequelae nor predispose the patient to future seizure activity (10). However, intra-session seizures do pose a risk of injury within the chamber, especially in veterinary medicine where monoplace chambers are most common and the patient cannot be reached immediately. In monoplace chambers, a patient is enclosed in a small chamber, and the entire chamber is pressurized and subjected to the rise in partial pressure of oxygen. This is in contrast to multiplace chambers available in human medicine, where a room containing multiple patients and nurses is pressurized, and individual patients receive supplemental oxygen through masks.

The objectives of this study were to evaluate the clinical use and treatment parameters of HBOT in small animals at a university teaching hospital and to report the frequency of adverse events, with particular interest in the frequency and potential risk factors for CNS oxygen toxicity. The study findings will provide foundational knowledge regarding potential veterinary indications and complications of HBOT and guide future prospective, randomized controlled trials to further evaluate its use in veterinary medicine.

## Materials and Methods

The electronic medical records of all small animal patients receiving HBOT were collected from a university teaching hospital over the period from November 2012 to February 2020. All hyperbaric sessions were performed in a monoplace chamber with three patient-viewing windows and three-view, live-feed video (Hyperbaric Veterinary Medicine, Boca Raton, Florida), with sessions monitored at all times by a veterinarian or veterinary technician certified in chamber use. Chamber certification was provided for each chamber operator by the chamber manufacturer after completion of an online training program and a series of treatment sessions performed with supervision from an individual experienced in the use of HBOT. Data collected included patient species, breed, age, weight, indication for treatment, total number of sessions, details of session parameters [maximum pressure, oxygen flow, fraction of inspired oxygen (FiO_2_), time at pressure], and occurrence of adverse events. Adverse events were characterized as minor if the HBOT treatment session could be safely continued and no additional monitoring or care was required following the event. Adverse events were characterized as major if the HBOT treatment session was discontinued and alterations were made to subsequent HBOT sessions or additional supportive care was required following the event. Both focal and generalized seizures which occurred during an HBOT session were suspected to be due to CNS oxygen toxicity. A focal seizure was defined as spastic and repetitive movement of the patient localized to a portion of the body during which the patient continued to be responsive to the HBOT operator, but which did not stop upon a distraction (e.g., turning to look when operator tapped on the window). A generalized seizure was defined as spastic movement affecting the entire body during which the patient was unresponsive to distraction, and which was followed by a post ictal period during which the patient demonstrated an abnormal depressed mentation. For both focal and generalized seizures, these were attributed to CNS oxygen toxicity if the signs improved upon reduction in chamber pressure. Patients were not excluded from receiving HBOT if they had a history of prior seizures at the discretion of the clinician overseeing treatment.

For statistical analysis, the mean, standard deviation, and range are provided for descriptive data. Further statistical analysis was not performed on special species due to limited data points. The remaining data were assessed for normality, and a Kruskal-Wallis test performed on non-parametric group comparison data with a *p*-value set at 0.05 for significance. For CNS oxygen toxicity treatment data, univariate analysis was performed with variables of interest; variables with a *p* < 0.2 were included in the initial multivariable logistic regression model with backwards elimination of variables with *p* > 0.1 to achieve the final model. For assessment of risk for CNS oxygen toxicity, the dependent variable utilized in the regression model was occurrence of CNS oxygen toxicity, with independent variables including gender, age, weight, neurologic indication (yes/no), total number of treatment sessions, and species (dog/cat). Statistical analysis was performed in Microsoft Excel 2016 using the Data Analysis ToolPak.

## Results

During the retrospective study period, 2,792 HBOT sessions were performed; patients were primarily dogs (*n* = 542, 94.1%) with few cats (*n* = 24, 4.2%) and other species (*n* = 10, 1.7%). Four dogs received HBOT for two clinically distinct indications within the study period.

Dogs in the study accounted for 2,632 (94.1%) HBOT sessions. Mean age of dogs was 6.7 ± 3.8 years (range 0.2–16), weighing 18.3 ± 13.2 kg (range 1–71). Dogs received on average 4.9 ± 8.3 sessions (range 1–93). There was no difference in number of sessions between species (*p* = 0.40). Data demonstrating number of dogs and sessions for different indications are available in Tables 1 and 2. There was a significant difference in the average number of sessions per patient when compared to the clinical indication for pursuing HBOT, with dogs diagnosed with oomycete infections receiving a greater number of treatment sessions than dogs with other indications (*p* < 0.001). Protocols used during chamber sessions varied with pressures reported up to 3 atmospheres absolute (ATA). A majority (88.5%) were performed at 2 ATA; pressure was gradually increased to the desired therapeutic level over approximately 15 min. Time at pressure ranged from 30 to 75 min with a majority of treatments spending 45 min at pressure. The chamber was then decompressed gradually over approximately 15 min to 1 ATA (sea level) prior to patient removal.

**Table 1:** Patient and treatment characteristics of dogs receiving hyperbaric oxygen therapy categorized by treatment indication.

**Treatment indication**	**No. of patients [%]**	**Mean age**	**Mean weight**	**No. of**	**Mean sessions**
		**± SD (years)**	**± SD (kg)**	**sessions [%]**	**± SD [range]**
Neurologic	273 [50.4]	6.4 ± 3.3	14.4 ± 12.1	974 [37.0]	3.6 ± 5.4 [1–77]
Tissue healing	170 [31.4]	6.8 ± 4.0	21.2 ± 12.7	695 [26.4]	4.1 ± 5.8 [1–58]
Oomycosis	30 [5.5]	2.6 ± 1.6	32.1 ± 10.1	526 [20.0]	17.5 ± 16.1 [1–59]^*^
Neoplasia/Radiation	29 [5.4]	10.9 ± 2.2	23.7 ± 13.9	171 [6.5]	5.9 ± 6.4 [1–29]
Miscellaneous	40 [7.4]	8.0 ± 4.6	18.9 ± 13.4	266 [10.1]	6.7 ± 15.3 [1–93]

* *p < 0.001 compared to all other indications*.

**Table 2:** Patient and treatment characteristics of dogs receiving hyperbaric oxygen therapy for miscellaneous indications (*n* = 40 patients from Table 1).

**Treatment indication**	**No. of patients**	**Mean age**	**Mean weight**	**Total no. of**
		**(years)**	**(kg)**	**sessions [range]**
Vascular event/Thromboembolism	10	10.2	19.6	30 [1–10]
Sago palm toxicity	6	1.6	10.7	24 [3–5]
Osteoarthritis	5	12.2	22.8	59 [1–37]
Pancreatitis	5	8.8	13.9	10 [1–3]
Sepsis	4	7.3	36.2	10 [1–5]
Aspergillosis	2^*^	6.5	21.5	96 [1–93]
Carbon monoxide toxicity	2	0.6	15.6	6 [2–4]
Cognitive dysfunction	2	12.3	21.0	57 [1–7]
Urinary tract infection	2	13.3	24.3	6 [3]
Immune-mediated hemolytic anemia	1	6.0	16.5	1 [n/a]
Post-cardiac arrest	1	4.0	30.7	18 [n/a]

* *n = 1 dog each treated for systemic and sinonasal form of aspergillosis*.

Cats accounted for 124 (4.2%) HBOT sessions. The mean age of cats was 8.8 ± 5.4 years (range 0.4–17), which was significantly greater than that for dogs (*p* = 0.04). Mean weight was 4.9 ± 2.2 kg (range 1.4–10.1). Cats received on average 5.2 ± 6.0 sessions (range 1–26). The most common treatment indication was for wounds or tissue healing (*n* = 15, 62.5%), followed by thrombus or vascular events (*n* = 4, 16.7%), urinary obstruction (*n* = 4, 16.7%), and intervertebral disc disease (*n* = 1, 4.2%).

Other species consisted of a variety of pocket pets and exotics including two macaws and one each of a capuchin monkey, opossum, bat, gray squirrel, skunk, iguana, and goat accounting for 36 treatment sessions, and an average of 3.6 ± 2.6 sessions per patient (range 1–10). The most common indication for HBOT for these patients was for external wounds or abscesses (*n* = 7, 70%), with one patient each receiving HBOT for myelopathy, gastrointestinal perforation, and concurrent pneumonia and myositis.

Minor reported side effects were rare in dogs and were limited to perceived agitation (*n* = 2) and vomiting (*n* = 3). No adverse events were noted during sessions with cats or other species.

The most common major adverse effect noted during HBOT sessions was attributed to CNS oxygen toxicity. Signs of CNS oxygen toxicity occurred in 19 dogs (3.5% of dogs) with an incidence rate of 0.7% accounting for all treatment sessions (Table 3). These signs included both focal seizures (*n* = 8, 0.3% of all sessions) and generalized seizures (*n* = 11, 0.4% of all sessions). Central nervous system oxygen toxicity occurred during the first session in 6/19 dogs while the remaining 13 dogs experienced CNS oxygen toxicity only after multiple uneventful treatment sessions (session 3–14). Duration of treatment at target chamber pressure at the time of onset of signs of CNS oxygen toxicity was variable and was recorded for 16/19 cases. Two patients experienced signs during initial pressurization and 1 dog after 16 min at pressure. The remaining 13 patients displayed onset of signs between 25 min at pressure and the end of treatment (with total treatment duration being 45 min except for one dog which received a 60-min treatment). Atmospheric pressure was recorded in the patient's record in 18/19 cases; 14 cases were at 2 ATA, with two dogs showing signs of focal seizures during pressurization beginning around 1.1–1.5 ATA, and one dog each at 2.2 and 2.7 ATA. In all cases of suspected CNS oxygen toxicity, the patient spontaneously recovered upon return to normal pressure and no long-term effects were reported. In 9 of the 19 cases, additional treatment sessions were performed at a lesser atmospheric pressure than that at which the seizures occurred, with no further adverse events reported. Univariable analysis identified gender and age for inclusion in the multivariate regression model (Table 4). Increasing age of the dog and gender was found to be significantly associated with risk of CNS oxygen toxicity. Dogs over 6 years of age were 4.6 times more likely to have signs of CNS oxygen toxicity (*p* = 0.01). Female dogs were 2.4 times more likely to have signs of CNS oxygen toxicity compared to males (*p* = 0.01) (Table 5).

**Table 3:** Patient and treatment characteristics for dogs with suspected CNS oxygen toxicity during hyperbaric oxygen therapy.

**Patient**	**Age (years)**	**Weight (kg)**	**Sex**	**Generalized or focal seizure**	**Treatment indication**	**Session#**	**Timing (min) (of 45 unless otherwise noted)**	**Pressure (ATA)**	**Medications**
1	11.0	1.0	SF	Generalized	Post cardiac arrest, cervical disc herniation	14 of 20	35	2.7	Not reported
2	14.0	21.3	M	Focal	Wound	4 of 10	During pressurization	1.5	Tramadol, trazodone
3	8.0	12.0	SF	Focal	Thrombo-embolism	1 of 1	40	2.2	Clopidogrel, aspirin, dalteparin, enalapril, ondansetron, amlodipine
4	7.0	7.6	SF	Generalized	Spinal trauma	5 of 5	Not reported	Not reported	Fentanyl, tramadol, sulfadimethoxine ormetoprim
5	1.0	6.2	NM	Focal	IVDD	6 of 6	55 of 60	2	Tramadol, carprofen
6	4.0	4.6	SF	Generalized	IVDD	3 of 3	16	2	Tramadol, prazosin, prednisone, gabapentin
7	10.0	10.0	NM	Focal	Wounds	8 of 13	25	2	Ampicillin-sulbactam, marbofloxacin, acepromazine, tramadol
8	15.0	8.1	SF	Generalized	Thrombo-embolism	1 of 1	25	2	Methadone, prednisone, diphenhydramine, pantoprazole, clopidogrel, aspirin, dalteparin, trimethoprim sulfamethoxazole
9	14.0	24.7	SF	Generalized	Post-op edema	5 of 18	37	2	Methadone, trazodone, carprofen, cephalexin
10	8.0	35.0	NM	Focal	IVDD	6 of 7	44	2	Tramadol, trazodone, carprofen, prazosin
11	13.0	32.5	SF	Generalized	T3–L3 myelopathy	1 of 2	40	2	Tramadol, gabapentin, firocoxib, levothyroxine
12	11.0	11.4	SF	Generalized	IVDD	3 of 3	44	2	Methadone, ursodiol, levothyroxine, trilostane, gabapentin, cefazolin, prazosin
13	1.0	12.4	F	Focal	Vasculitis	10 of 18	During pressurization	1	Mycophenolate, prednisone, pentoxyfylline, trazodone, dexmedetomidine, atipamezole
14	9.5	13.7	NM	Generalized	Post-op edema	1 of 1	Not reported	2	Carprofen, gabapentin, trazodone
15	12.0	21.6	SF	Focal	Snake bite	1 of 1	35	2	Cerenia, ampicillin-sulbactam, ondansetron, imipenem, capromorelin
16	6.0	25.5	SF	Generalized	IVDD	3 of 3	44	2	Methadone, gabapentin, amantidine, carprofen, cerenia, trazodone
17	7.5	45.8	NM	Focal	Wound	3 of 3	Not reported	2	Clindamycin, gabapentin
18	11.0	23.5	SF	Generalized	Wound	4 of 7	30	2	Nitrofurantoin, carprofen, tramadol
19	7.0	14.3	SF	Generalized	ANNPE	1 of 3	39	2	Prednisone, trazodone

*M, intact male; NM, neutered male; F, intact female; SF, spayed female; IVDD, intervertebral disc disease; ANNPE, acute non-compressive nucleus pulposus extrusion; ATA, atmospheres absolute*.

**Table 4:** Univariate analysis of comparison of patient characteristics and number of treatment sessions between occurrence of CNS oxygen toxicity.

	**CNS oxygen toxicity**	**No CNS oxygen toxicity**	***p*** **-Value**	**All patients**
	**(*n* = 19)**	**(*n* = 544)**		**(*n* = 563)**
Gender				
Male	6	288	0.01	294
Female	13	256		269
Age				
<6 years	4	299	0.01	303
≥6 years	15	245		260
Weight (kg)	17.43 ± 11.65	17.80 ± 13.3	0.82	17.80 ± 13.25
Neurologic indication				
Yes	10	265	0.69	273
No	9	279		290
No. treatment sessions	6.37 ± 6.35	4.80 ± 8.26	0.36	4.90 ± 8.20
Species				
Dog	19	520	0.31	539
Cat	0	24		24

**Table 5:** Results of final multivariable regression model showing effect of patient sex and age on occurrence of CNS oxygen toxicity.

**Variable**	**Odds ratio**	***p*** **-Value**
	**(95% confidence interval)**	
Gender	2.4 (0.9–6.5)	0.01
Age	4.6 (1.5–14.0)	0.01

A single dog developed pulmonary edema following HBOT; this may have been a manifestation of pulmonary oxygen toxicity to the lungs or may have been a natural sequelae of the patient's disease process or iatrogenic fluid overload. This dog had been diagnosed with diffuse discospondylitis and aspiration pneumonia a week prior to treatment, and a single HBOT session was elected following surgery for pyometra. The dog developed worsening respiratory signs later in the day after receiving HBOT, and thoracic radiographs were concerning for the development of pulmonary edema. The dog received a single dose of furosemide, intravenous fluids were discontinued, and the respiratory signs resolved.

## Discussion

With 2,792 treatment sessions reported here, this is to date the largest collection of data regarding the use of HBOT for a variety of clinical indications across several veterinary species. Common indications included neurologic injury, especially acute myelopathies, wound or tissue healing, oomycete infection control, neoplasia, and a number of miscellaneous conditions based on common clinical indications in people or evidence from research models supporting its use.

Only one other study utilizing HBOT for multiple indications is available for comparison to the data presented here regarding common treatment indications for HBOT in small animal veterinary patients, with some similarities and differences (17). In that study, neurologic and integument disorders were similarly common treatment indications. However, while intervertebral disc disease made up the vast majority of neurologic cases in this study, 9/29 patients treated in the Birnie et al. study had intracranial disease (17). Similarly, while wounds were the most common integument disorder treated here with skin flaps and grafts representing a small population of treated patients, HBOT was more commonly utilized following skin flaps and grafts rather than for traumatic wound care in the study by Birnie et al. (17). In addition to varying clinical indications, chamber types and treatment parameters vary across studies. The chamber type utilized here was a veterinary-specific monoplace chamber, while previous studies have used different veterinary or human-intended HBOT chambers (7–9, 17). These chambers may have different output parameters such as variable oxygen flow rates and exchange of CO_2_, temperature or humidity regulation, or maximal pressurization capacities with unknown effects on treatment outcomes. Within the population reported in this study, treatment parameters varied, although most treatments were performed at 2 ATA for 45 min at pressure, which is comparable to other studies (16, 17).

This study provides valuable data regarding potential adverse events and in particular that of CNS oxygen toxicity. The occurrence of CNS oxygen toxicity was in contrast to a recent study in which no signs of CNS oxygen toxicity or major adverse events occurred over 230 treatment sessions (17). In that study, treatments were performed at 2 ATA for 45 or 60 min. While treatment pressure and times were variable in the study reported here, CNS oxygen toxicity primarily occurred at 2 ATA within a 45-min treatment duration. The studies utilized different veterinary-specific monoplace chambers; it is unknown what, if any, effects chamber design has on CNS oxygen toxicity. The effects of CNS oxygen toxicity did not appear to be due to cumulative response alone, as many dogs underwent dozens of sessions without complications, and no further events were noted in those patients who continued to receive treatment following a major adverse event.

The pathophysiology of CNS oxygen toxicity is not fully understood. One thought implicates oxidative stress and the generation of free radicals beyond that which can be counteracted by the protective endogenous antioxidant capabilities of the body. Hyperoxia results in increased generation of reactive oxygen species (ROS) which can alter membrane lipid peroxidation and enzymatic inhibition in the brain (18, 19). Regulation of cell excitability may also be altered by ROS (19). Rising levels of nitric oxide have also been implicated through its actions as a vasodilator of cerebral vessels, and as a neurotoxic and pro-oxidative free radical (19, 20).

Elevated partial pressures of carbon dioxide have also been implicated as a factor associated with CNS oxygen toxicity (21). Carbon dioxide toxicity in humans is expected with prolonged exposure at >1,000 ppm, with signs such as increased heart rate, respiratory rate, and fatigue (22). While not prospectively recorded during treatment sessions in this study, CO_2_ was monitored by the attending staff during all treatments using a portable monitoring system with a sensor placed in the chamber. Elevated CO_2_ levels were combated with increasing O_2_ flow to the chamber; anecdotally, partial pressures may temporarily reach 3,000–4,000 ppm during treatments with large, heavily panting dogs. Given that dog size was not a factor associated with increased risk of CNS oxygen toxicity, we suspect that elevated CO_2_ does not play a role in this adverse effect of HBOT.

In human medicine, medications lowering the seizure threshold have been suspected to contribute to CNS oxygen toxicity, although a definitive association between the use of these medications and seizures during HBOT has not been found. Commonly implicated medications include tricyclic antidepressants and selective serotonin reuptake inhibitors, antibiotics such as cephalosporins, tramadol, and narcotics (21, 23, 24). In this study, a majority of the dogs developing signs of oxygen toxicity were receiving at least one medication which can lower the seizure threshold, with tramadol and trazodone the most commonly utilized medications. It is a limitation of the study that medications received while undergoing HBOT was not collected on all patients for comparison among groups. While not specifically assessed here, it is the authors' clinical impression that many of the patients undergoing HBOT were receiving these medications and a vast majority did not have signs of oxygen toxicity. Individual sensitivities to the effects of such single medications or combination therapeutics with hyperbaric oxygen remain challenging to evaluate. However, further prospective studies assessing medication use, dosages, and timing of administration in relation to HBOT could prove valuable in identifying a relationship between various medications and oxygen toxicity.

It is unclear why age was a risk factor for CNS oxygen toxicity, although this is consistent with findings in people (25). It is hypothesized that with age, there is a reduced tolerance for oxidative stress and accumulation of reactive oxygen and nitrogen species (25). It is also possible that the combination of the effects of aging and the use of medications previously discussed may together lower the seizure threshold and increase risk of oxygen toxicity (22). The association with the female sex is also consistent with reports in people as well as laboratory rats. It has been proposed that females are at greater risk due to higher concentrations of endogenous estrogen, which can have proconvulsant properties (25, 26). However, as most of the female dogs affected in this study were spayed, this suggests there may be another as yet unknown cause of risk in females. Central nervous system oxygen toxicity and other potential adverse effects of HBOT should be discussed with all owners prior to initiating this therapy, and it is reasonable to consider treatment at lower pressures in older and/or female dogs. However, given the overall low incidence of CNS oxygen toxicity seen in this study and the self-limiting nature of the signs, the authors do not feel increased age nor female gender precludes treatment at standard parameters with careful monitoring.

The CNS oxygen toxicity rate identified in this population of dogs was higher than that typically identified in humans. This may be due to the higher metabolic rate in dogs compared to people. It is also interesting to note that no signs of CNS oxygen toxicity were identified in cats in this study. A previous study identified minor adverse events in cats including head shaking and lip smacking, but similarly identified no major complications in felines (17). The lack of major adverse events in cats and other species may have been due to the relatively few number of cats and other species treated compared to dogs, or could be due to an inherent resistance to CNS oxygen toxicity.

Several species other than dogs and cats were represented in this study, with wound healing as the primary indication for utilization of HBOT and no adverse events seen. While the retrospective data did not provide information sufficient to determine the clinical benefit of HBOT, it appeared safe and could be considered as a treatment option for pocket pets and exotics.

A major limitation of this study was the retrospective nature and inherent barriers to data extraction from a patient medical record. While major adverse events were clearly noted in records, it is likely that minor adverse events (e.g., vomiting) were underreported, especially as they may have occurred following the conclusion of the treatment session and their occurrence not associated with recent hyperbaric therapy.

The collected data provide information on the variety of conditions for which HBOT was utilized in a veterinary university teaching hospital. Adverse events were rare, with signs of CNS oxygen toxicity occurring most commonly. The signs of CNS oxygen toxicity were limited to a very small population of patients, were self-limiting, and did not recur in subsequent hyperbaric therapy sessions. Thus, this study suggests that HBOT is safe for use across a variety of clinical indications, in various species, and with repeated treatment sessions. However, increasing age was identified as a risk factor for CNS oxygen toxicity, and females were 2.4 times more likely to have CNS oxygen toxicity. Further prospective, randomized, controlled trials should evaluate specific clinical indications and outcomes, to determine those patients that receive the most benefit from HBOT, as well as the ideal protocol for chamber use with attention to potential risk factors to CNS oxygen toxicity and other adverse events.

## Data Availability Statement

The datasets presented in this study can be found in online repositories. The names of the repository/repositories and accession number(s) can be found below: Figshare Data Repository - https://doi.org/10.6084/m9.figshare.13244507.v1.

## Author Contributions

CM, CK, LE and JS contributed in formulation of the study idea. CM and CK collected data. CM and JS performed statistical analysis and drafted the manuscript. All authors contributed to the manuscript preparation and revision.

## Conflict of Interest

CK was employed by company Whole Animal Gym. The remaining authors declare that the research was conducted in the absence of any commercial or financial relationships that could be construed as a potential conflict of interest.

## Publisher's Note

All claims expressed in this article are solely those of the authors and do not necessarily represent those of their affiliated organizations, or those of the publisher, the editors and the reviewers. Any product that may be evaluated in this article, or claim that may be made by its manufacturer, is not guaranteed or endorsed by the publisher.
